# Prevalence, symptoms and management of uterine fibroids: an international internet-based survey of 21,746 women

**DOI:** 10.1186/1472-6874-12-6

**Published:** 2012-03-26

**Authors:** Anne Zimmermann, David Bernuit, Christoph Gerlinger, Matthias Schaefers, Katharina Geppert

**Affiliations:** 1 Charité - Universitätsmedizin Berlin, Berlin School of Public Health, Seestraße 73, Berlin 13347, Germany; 2 Bayer Healthcare Pharmaceuticals, Berlin 13342, Germany

## Abstract

**Background:**

In 2009 the Uterine Bleeding and Pain Women's Research Study (UBP-WRS) was conducted interviewing 21,479 women across 8 countries in order to gain patient-based prevalence data on uterine pain and bleeding indications and investigate uterine symptoms and women's treatment experiences. This article shows relevant results of the study for the indication uterine fibroids providing data on self-reported prevalence, symptomatology and management of uterine fibroids.

**Methods:**

2,500 women (USA: 4,500 women) in each country (Brazil, Canada, France, Germany, Italy, South Korea, the UK, the USA) completed an online survey. Women included were in their reproductive age (age group 15-49 years; USA: 18-49 years) and had ever experienced menstrual bleedings. Quotas were applied for age, region, level of education and household income of respondents. Variables have been analyzed descriptively and exploratory statistical tests have been performed.

**Results:**

The self-reported prevalence of uterine fibroids ranged from 4.5% (UK) to 9.8% (Italy), reaching 9.4% (UK) to 17.8% (Italy) in the age group of 40-49 years. Women with a diagnosis of uterine fibroids reported significantly more often about bleeding symptoms than women without a diagnosis: heavy bleedings (59.8% vs. 37.4%), prolonged bleedings (37.3% vs. 15.6%), bleeding between periods (33.3% vs. 13.5%), frequent periods (28.4% vs. 15.2%), irregular and predictable periods (36.3% vs. 23.9%). Furthermore women with diagnosed uterine fibroids reported significantly more often about the following pain symptoms: pressure on the bladder (32.6% vs. 15.0%), chronic pelvic pain (14.5% vs. 2.9%), painful sexual intercourse (23.5% vs. 9.1%) and pain occurring mid-cycle, after and during menstrual bleeding (31.3%, 16.7%, 59.7%, vs. 17.1%, 6.4%, 52.0%). 53.7% of women reported that their symptoms had a negative impact on their life in the last 12 month, influencing their sexual life (42.9%), performance at work (27.7%) and relationship & family (27.2%).

**Conclusions:**

Uterine fibroid is a common concern in women at fertile age causing multiple bleeding and pain symptoms which can have a negative impact on different aspects in women's life.

## Background

Uterine fibroids (myomas or leiyomymas) are benign, monoclonal tumors of the smooth muscle cells found in the human uterus [1,2]. Despite the fact that their cause is still unknown, yet there is considerable evidence that estrogens and progestogens proliferate tumor growth [3,4], as the fibroids rarely appear before menarche [5] and regress after menopause [6]. Uterine fibroids are the most common benign tumors in women and the leading indication for hysterectomies in the USA [7,8], nevertheless, epidemiological data on fibroid prevalence and incidence are limited and reliable population-based research is lacking [9]. Available data are difficult to compare due to differences in the study population and screening methods. Prevalence data range from 5% to 21% [10-13]. In a US study with randomly selected women between 35 to 49 years, who were screened by self-report, medical record and sonography, the incidence of uterine fibroids by age 35 was 60% among African-American women, increasing to > 80% by age 50, whereas Caucasian women showed an incidence of 40% by age 35, and almost 70% by age 50 [14]. Apart from race, other possible risk factors for developing uterine fibroids are early age at menarche [15], familial predisposition [16] and overweight [17,18]. Parity [15,19-21] and smoking may protect from developing myomas [17,19,20,22].

The majority of women with uterine fibroids are asymptomatic, consequently get less clinical attention and fibroid tumors often remain undiagnosed [23,24]. Symptomatic women typically complain about abnormal uterine bleeding, specifically in terms of heavy and prolonged bleeding [25]. In a study of Wegienka et al. (2004) women with myomas were more likely to report a "gushing"-type of bleeding and high pad/tampon use than women without myomas [26]. Additionally, women with uterine fibroids may suffer more often from dyspareunia and non-cyclic pelvic pain [27]. Although bleeding and pelvic pain symptoms are frequently reported in literature as main symptoms related to uterine fibroids, the number of systematic studies on fibroid symptoms is limited.

Therapeutic options to treat these symptoms include medical therapy, surgical interventions and uterine artery embolization [28]. Medical treatments used to manage bleeding symptoms are oral contraceptives or progestins, although there is no evidence for their efficacy in treating myomas [29]. Other medical alternatives are the Levonorgestrel-releasing intrauterine system (LNG-IUS) and GnRH-agonists. However, the LNG-IUS cannot be applied in case of significant distortion of the uterine cavity and the duration of treatment with GnRH-agonists is limited by the induction of hypoestrogenic symptoms. According to the reproductive desire of the patient and the severity of symptoms surgical procedures comprise myomectomy, endometrial ablation or hysterectomy.

In 2009 the Bayer Healthcare Pharmaceuticals initiated the Uterine Bleeding and Pain Women's Research Study (UBP-WRS), an internet-based-study across 8 countries. The objective of this large study was to gain patient-based prevalence data on uterine pain and bleeding indications and investigate uterine symptoms and women's treatment experiences. This article shows relevant results of the study for the indication uterine fibroids. According to our knowledge it is the largest international population-based study available on self-reported prevalence, symptomatology and management of uterine fibroids. It allows for cross country-comparison regarding prevalence data and management of uterine fibroids and reveals differences in symptomatology and risk factors in comparison with women without a diagnosis of uterine fibroids.

## Methods

### Recruitment and study population

The UBP-WRS is a cross-sectional study using an online-method approach. The online survey was conducted during November and December 2009 across 8 countries including Brazil, Canada, France, Germany, Italy, South Korea, the UK and the USA. In order to reach appropriate potential participants of women in a representative manner across countries, study participants were recruited via an online-panel. The panel is hosted by the online market research service provider Global Market Insite (GMI), a certified member of the European Society for Opinion and Marketing Research (ESOMAR). Panelists are recruited from more than 500 sources, such as web advertising or permission-based databases and sign up to participate by filling out a comprehensive online registration form. In a second step panelists activate their account by clicking a link sent to them via email immediately after registration. Panel information is updated on a regular basis throughout the year and panel members receive a maximum of 4 different surveys per month across a variety of topics and in their native languages.

Inclusion criteria were: women in their reproductive age (age group 15-49 years, except USA: 18-49 years due to legal restrictions) who have ever experienced menstrual bleedings. In order to collect a cohort of women representative of the general population, quota limits within each country based on available national statistics or census data were enforced for age, geographic region, level of education and household income. Within-country population limits for data acquisition were relaxed after 80% of the sample size had been achieved.

Institutional Review Board (IRB)/Ethics Committee decided approval was not required for this study.

### Data collection

2,500 women per country (USA: 4,000 women) meeting the inclusion criteria and the quotas were enrolled in the study. The survey instrument was a self-administered online-survey written in English and translated in Spanish, Portuguese, German, Italian, French and Korean, accounting for local language characteristics. The survey took approximately 45 minutes to complete and collected data in a number of categories: demographics, patient characteristics, aspects of the menstrual cycle, health care visits, diagnosed conditions, symptoms, treatments received, surgeries and impact on life. Those women, who answered that their doctor or another health professional has ever told them the diagnosis of uterine fibroids, form the uterine fibroid population in this study. The comparison group is defined as those women who did not receive a diagnosis of uterine fibroids from their doctor or health professional. The survey also included questions designed to identify gynecological conditions other than uterine fibroids, such as endometriosis. These findings will be reported elsewhere [30].

### Statistical methods

All variables were analyzed using descriptive statistical methods, giving the number of non-missing observations, mean, and standard deviation for metric variables and absolute and relative frequency counts for categorical variables. Observations were age-weighted within each country according to census data. Missing response items were not imputed.

Exploratory statistical tests were performed to test the null hypothesis that there was no difference between women with and without a diagnosis of uterine fibroids. The χ^2^-test was used for categorical data and analysis of variance for metric data. In addition, logistic regression was applied to analyze the influence of age and co-morbidities like endometriosis and adenomyosis in addition to the occurrence of uterine fibroids. All statistical tests used a significance level α of 5%. As appropriate for exploratory analyses, no corrections for multiple testing were performed. 95-% Confidence Intervalls (CI) for the proportions were calculated using the exact Clopper-Pearson method. The statistical analyses were performed using SPSS software.

## Results

### Study participation

In total, 251,181 women were invited to participate in the survey, of which 14.6% (n = 36,770) entered screening; a further 11,607 (4.6%) were filtered out during the screening stage. Of the remaining population (n = 25,163), 1.4% (n = 3,414) of all women invited either did not complete the survey and were excluded, or were eliminated by quality control procedures. Between 2,500 and 2,558 surveys were completed by participants in each of the eight countries, with the exception of the USA, in which 4,039 surveys were submitted (see Table 1). The final survey population used in data analysis comprised 21,746 women aged 15-49 years (USA: age range 18-49 years).

**Table 1:** Study participation per country

	No. of women per country							
	**France**	**Germany**	**Italy**	**UK**	**USA**	**Canada**	**Brazil**	**South Korea**

**invited**	25,973	16,743	19,564	41,840	78,567	32,589	26,905	9,000

**started screening**	4,617	4,392	4,276	5,179	5,253	4,272	5,510	3,271

**meeting screening criteria and quota**	2,954	2,939	2,815	2,902	4,495	3,194	3,210	2,654

**completed survey**	**2,543**	**2,558**	**2,519**	**2,500**	**4,039**	**2,514**	**2,552**	**2,524**

### Self-reported prevalence

**Figure 1:**
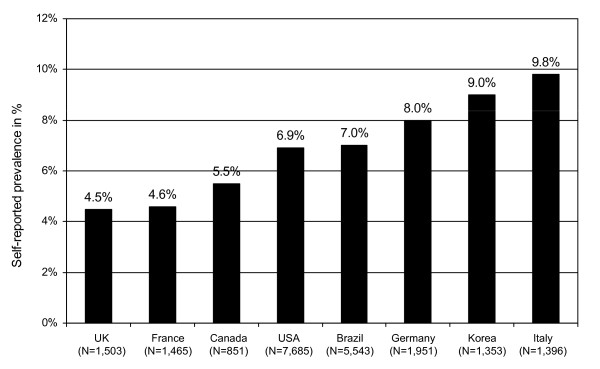
**Self-reported prevalence of uterine fibroids across countries**.

The self-reported prevalence of uterine fibroids ranged from a low of 4.5% in UK and 4.6% in France to a high of 9.8% in Italy and 9.0% in Korea (see Figure 1). Although prevalence increased with age, already 1.8% of the 20 to 29 year old women received a diagnosis (see Table 2). Self-reported prevalence in the age group of 40 years and older rose to 14.1% across all countries, ranging from 9.4% in UK (n = 45) to 17.8% in Italy (n = 86).

**Table 2:** Self-reported prevalence of uterine fibroids by age groups

		Women by age group		
	15 to 19 years*	**20 to 29 years**	**30 to 39 years**	**40 to 49 years**

**Total N**	2,180	6,335	6,378	6,853

**Self-Reported Prevalence in % (N)**	0.4 (8)	1.8 (115)	7.0 (447)	14.1 (963)

*Age range in the USA: 18 to 49 years

### Risk Factors

With a mean age of 40.4 ± 6.9 years women who reported a diagnosis of uterine fibroids (n = 1,533) were in average 8 years older than women without the diagnosis (mean age 32.4 ± 9.6 years; n = 20,213; p < 0.001). There was no statistical difference in the mean age at menarche between both groups (diagnosed women with uterine fibroids: 12.9 ± 1.9 years; undiagnosed women: 12.9 ± 2.1 years; p = 0.220). The percentage of women having children was higher among women with diagnosed uterine fibroids (67.1%) compared to women without a diagnosis (46.2%). After adjusting for age, this difference disappears.

### Bleeding Symptoms

The frequency of uterine bleeding symptoms of women who reported a diagnosis of uterine fibroids in comparison to women without a diagnosis of uterine fibroids is shown in Table 3. About 59.8% of women with a uterine fibroid diagnosis experienced heavy bleedings compared to 37.4% of women without a diagnosis (p < 0.001), followed by prolonged bleedings, which 37.3% of women with uterine fibroids indicated compared to 15.6% of undiagnosed women (p < 0.001). Furthermore, diagnosed women with uterine fibroids reported more often bleedings between periods and frequent periods which occur more often than just every 24 days (33.3% and 28.4% vs. 13.5% and 15.2%), (p < 0.001), as well as irregular and unpredictable periods (36.3% vs. 23.9%), (p < 0.001). There was minor difference for women with a diagnosis of uterine fibroids regarding absent periods (p = 0.015) and infrequent periods (p = 0.048), which was defined as periods that occur less often than every 38 days. Multivariate analysis controlled for age did not show any difference in results, except that no statistical difference was found for shortened duration of menstrual bleeding (p = 0.099).

**Table 3:** Frequency of bleeding symptoms: Comparison of diagnosed women with uterine fibroids and women without a diagnosis of uterine fibroids

	Women with a diagnosis of uterine fibroids	Women without a diagnosis of uterine fibroids	p-value*
**Total N**	1,533	20,213	

**% of women with symptom**			

**Heavy menstrual bleeding**	59.8%	37.4%	p < 0.001

**Prolonged duration of menstrual bleeding**	37.3%	15.6%	p < 0.001

**Bleeding between periods**	33.3%	13.5%	p < 0.001

**Frequent periods **(i.e. periods occur more often than just every 24 days)	28.4%	15.2%	p < 0.001

**Irregular/unpredictable periods**	36.3%	23.9%	p < 0.001

**Light menstrual bleeding**	10.9%	18.7%	p < 0.001

**Absent periods**	13.9%	11.8%	0.015

**Infrequent periods **(i.e. periods occur less often than every 38 days)	16.7%	14.8%	0.048

**Shortened duration of menstrual bleeding**	13.1%	15.5%	0.012

*χ^2^-test applied

Two further questions supported the findings on bleeding symptoms: Of those women who indicated menstrual bleeding problems, women with diagnosed uterine fibroids reported a significantly longer duration of period (5.6 ± 3.1 days, n = 1,245) than women without a diagnosis of uterine fibroids (5.2 ± 2.4 days, n = 18,842), (p < 0.001). Additionally, women with uterine fibroids perceived their blood loss as heavier than women without uterine fibroids (mean 3.5 ± 1.0 vs. 3.2 ± 0.8) (p < 0.001), based on a 5-point-scale where 1 was defined as "very light" and 5 accordingly as "very heavy" blood loss during menstrual bleeding.

### Pain symptoms

Table 4 shows the frequency of different pain symptoms for women diagnosed with uterine fibroids compared to women without a diagnosis. Diagnosed women with uterine fibroids experienced significantly more often pressure on the bladder or inside the abdomen as well as chronic pelvic pain (32.6% and 14.5% vs. 15.0% and 2.9%) (p < 0.001). Pain at different points of time during the menstrual cycle was reported more commonly in women with uterine fibroids: They indicated to suffer significantly more often from pain occurring mid-cycle, after and during menstrual bleeding (31.3%, 16.7%, 59.7% vs. 17.1%, 6.4%, 52.0%) (p < 0.001). About 23.5% of women with uterine fibroids reported painful sexual intercourse compared to 9.1% of women without the diagnosis (p < 0.001). There was no statistical difference between both groups regarding menstrual cramps in the abdominal area just before menstrual period starts. In addition we conducted a logistic regression analysis controlled for age and the co-morbidities endometriosis and adenomyosis. Results showed high statistical significance (p < 0.001) for all pain symptoms listed in Table 4.

**Table 4:** Frequency of pain symptoms: Comparison of diagnosed women with uterine fibroids and women without a diagnosis of uterine fibroids

	Women with a diagnosis of uterine fibroids	Women without a diagnosis of uterine fibroids	p-value*
**Total N**	1,533	20,213	

**% of women with symptom**			

**Pressure on the bladder or inside the abdomen**	32.6%	15.0%	p < 0.001

**Chronic pelvic pain **(i.e. all or most days of the month)	14.5%	2.9%	p < 0.001

**Painful sexual intercourse**	23.5%	9.1%	p < 0.001

**Pain occurring mid-cycle/during ovulation **(approx. 10 days after the end of my period)	31.3%	17.1%	p < 0.001

**Pain after my menstrual bleeding or period**	16.7%	6.4%	p < 0.001

**Pain during menstrual bleeding or period**	59.7%	52.0%	p < 0.001

**Pain when going to the toilet**	10.8%	5.4%	p < 0.001

**Cramping during menstrual period**	50.2%	47.0%	0.014

**Menstrual/period cramps in the abdominal (belly) area just before menstrual bleeding starts**	48.7%	47.2%	0.246

*χ^2^-test applied

Those women who reported pain symptoms were asked about the severity of their symptoms in the last 12 month on a 10-point scale, where 1 was defined as "mild annoying pain" and 10 as "severe disabling pain" (see Table 5). Results show that women with diagnosed uterine fibroids score the severity of their pain symptoms significantly higher than women without a diagnosis, except for chronic pelvic pain where no difference between both groups was found. Severity scores among women with uterine fibroids were highest for cramping and pain during menstrual bleeding.

**Table 5:** Severity of pain symptoms in the last 12 month: Comparison of diagnosed women with uterine fibroids and women without a diagnosis of uterine fibroids

		Women with a diagnosis of uterine fibroids	Women without a diagnosis of uterine fibroids	p-value*
**Cramping during menstrual period**	N	527	8033	p < 0.001
	Mean ± SD	6.9 ± 2.4	6.2 ± 2.3	

**Pain during menstrual bleeding or period**	N	609	8315	p < 0.001
	Mean ± SD	6.7 ± 2.5	6.2 ± 2.3	

**Chronic pelvic pain **(i.e. all or most days of the month)	N	107	441	0.356
	Mean ± SD	6.7 ± 2.7	6.6 ± 2.4	

**Menstrual/period cramps in the abdominal (belly) area just before menstrual bleeding starts**	N	477	7429	p < 0.001
	Mean ± SD	6.3 ± 2.5	5.7 ± 2.4	

**Pain when going to the toilet**	N	106	807	0.002
	Mean ± SD	6.5 ± 2.4	5.7 ± 2.5	

**Pain after my menstrual bleeding/period**	N	138	796	p < 0.001
	Mean ± SD	6.3 ± 2.5	5.6 ± 2.4	

**Painful sexual intercourse**	N	211	1414	0.020
	Mean ± SD	6.2 ± 2.6	5.8 ± 2.4	

**Pressure on the bladder or inside the abdomen**	N	309	2308	p < 0.001
	Mean ± SD	5.9 ± 2.4	5.0 ± 2.4	

**Pain occurring mid-cycle/during ovulation **(approx. 10 days after the end of my period)	N	309	2581	p < 0.001
	Mean ± SD	5.8 ± 2.6	5.0 ± 2.5	

*χ^2^-test applied

### Management of uterine fibroids

**Figure 2:**
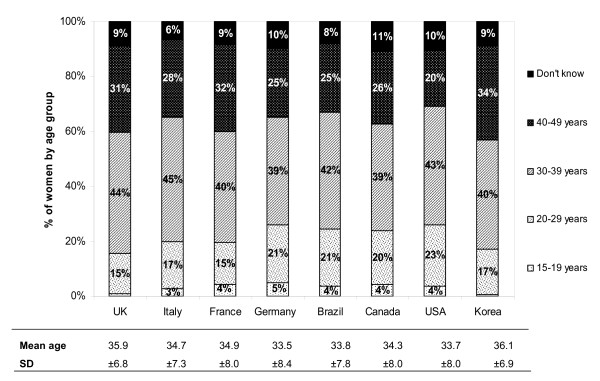
**Age at diagnosis across countries**.

The majority of women with uterine fibroids received their diagnosis in their thirties or forties. The mean age at diagnosis across all countries is similar, ranging from 33.5 ± 8.4 years in Germany to 36.1 ± 6.9 years in Korea (see Figure 2).

The percentage of women who received any medical treatments from their physician after the diagnosis of uterine fibroids varied from a low of 18.5% (95%CI: 12.7-25.4%) in Germany to a high of 55.8% (95%CI: 50.7-60.8%) in Brazil. The percentage of the remaining countries ranged between 39.4% (95%CI: 31.2-48.1%) (Italy) and 52.1% (95%CI: 47.7-56.4%) (USA). The types of medical treatment most frequently recorded were Oral Contraceptives (OCs), Painkillers and Iron Supplements. Oral Contraceptive usage was highest in Brazil (35.1%; 95%CI: 30.4-40.1%) and Italy (32.8%; 95%CI: 25.1-41.4%), whereas in Korea only 5.7% (95%CI: 2.3-11.5%) of women were treated with OCs. Painkillers for the treatment of uterine fibroid symptoms were most often used in the USA (34.7%; 95%CI: 30.7-38.9%), and least often in Italy (15.3%; 95%CI: 9.7-22.5%). Women in Canada reported the highest intake of Iron supplements (21.3%; 95%CI: 10.7-35.7%) compared to Brazil with the lowest percentage (9.8%; 95%CI: 7.0-13.2%). Besides, the following country specifics regarding medical treatment types commonly used were found: In UK as well as in France 10.3% (95%CI: 4.2-20.1%) of women with uterine fibroids were treated with an intrauterine system (Mirena^®^). Progestin treatment was quite common in Brazil (12.6%; 95%CI: 9.5-16.4%) and France (10.3%; 95%CI: 4.2-20.1%). About 17.3% (95%CI: 11.7-24.2%) of women in Germany and 17.0% (95%CI: 7.6-30.8%) of women in Canada indicated to use home remedies e.g. hot water bottle to treat uterine fibroids symptoms.

**Figure 3:**
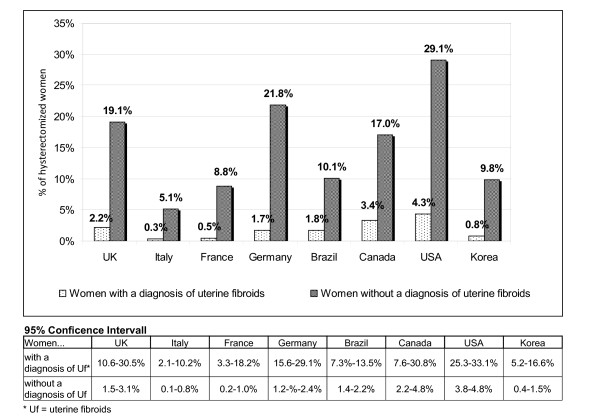
**Comparison of hysterectomized women with uterine fibroids andwithout uterine fibroids**.

In all countries, except for Brazil (47.7%; 95%CI: 42.6-52.8%), more than half of the women stated that they underwent surgical treatment for their uterine fibroids, ranging from 54.0% (95%CI: 45.3-62.6%) in Italy to 68.5% (95%CI: 64.3-72.4%) in the USA. Hysterectomy was the leading type of surgery in almost all countries; except for Italy and France where myomectomy is the most commonly reported surgical intervention (35.9% in France; 95%CI: 21.2-52.8%; 21.6% in Italy; 95%CI: 12.9-32.7%). As Figure 3 shows the percentage of hysterectomized women based on all women with uterine fibroids is highest in the USA with 29.1% (95%CI: 25.3-33.1%), followed by Germany with 21.8% (95%CI: 15.6-29.1) and the UK with 19.1% (95%CI: 10.6-30.5%) and lowest in Italy with 5.1% (95%CI: 2.1-10.2%). Furthermore the percentage of women who were hysterectomized was 5-fold (Canada) to 17-fold (France) higher in the population of diagnosed women with uterine fibroids compared to women without a diagnosis.

### Impact on Life

**Figure 4:**
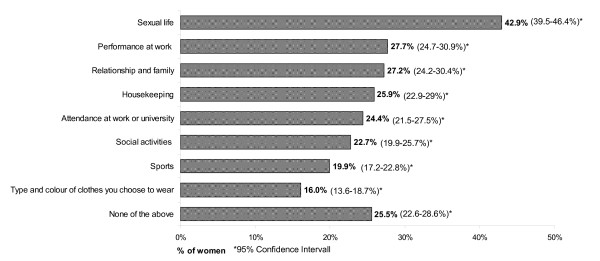
**Activities negatively affected by symptoms**.

Asking the women with diagnosed uterine fibroids (n = 1,533) about the impact of their symptoms in the last 12 month on their daily life, 14.8% (95%CI: 13.1-16.7%) of women reported a severe negative impact, 18.3% (95%CI: 16.4-20.4%) a moderate negative impact and 20.6% (95%CI: 18.6-22.7%) a mild negative impact. Almost 37.2% (95%CI: 34.8-39.7%) of diagnosed women answered that the symptoms do not have any impact on their daily life, whereas 9.0% (95%CI: 7.6-10.5%) did not know. There was no difference in the impact of symptoms on their daily life between the different age groups. Those women who reported a mild to severe impact of symptoms, where additionally asked which activities where negatively affected by their symptoms. About 42.9% (95%CI: 39.5-46.4%) of women stated that their sexual life was negatively affected, followed by performance at work (27.7%; 95%CI: 24.7-30.9%), relationship & family (27.2%; 95%CI: 24.2-30.4%) and housekeeping (25.9%; 95%CI: 22.9-29.0%) (see Figure 4).

## Discussion

### Scope of the survey

The UBP-WRS is among the largest online research endeavors to address women's healthcare issues. The survey directly recorded the experiences relating to uterine bleeding and pain of more than 21,000 women from diverse regional and demographic groups worldwide. This wealth of data has allowed the identification of various trends, specifically relating to the prevalence, symptoms and management of uterine fibroids.

### Limitations of the survey and methods

Nevertheless, a survey of this kind is subject to certain methodological limitations, which need to be carefully considered. First of all, the data may have been influenced by patient self-selection (self-selection bias) because of the fact that a) the responding women had access to and use the internet; b) in most cases they had experience in online market research participation in exchange for a small fiscal reward and c) they were willing to answer questions concerning their sexual health. Therefore, the study population can differ in their characteristics in comparison to the general female population. Representativeness of survey population regarding socio-demographic factors is only a minor risk due to the large sample size and applied quotas in age, level of education, country region and household income. All study results rely solely on women's self-reports and therefore on their subjective response. This is especially of concern regarding the data on diagnoses, symptoms and treatments because the results were not validated by medical records or clinical examinations. Subjective retrospective criteria, such as previous diagnosis and symptoms, can be biased as respondents may have difficulties to recall certain memories (recall bias). Finally, no linguistic validation of the translated questionnaire was conducted.

### Prevalence

The self-reported prevalence in this study ranged from 4.5% in UK to 9.8% in Italy. These results suggest that uterine fibroids are quite common among women in reproductive age, considering that the study population only includes diagnosed women with myomas, while the number of undiagnosed women remains uncertain. The results of the study are not directly comparable to other epidemiological studies due to the fact that the diagnosis has not been clinically proven by ultrasonography or other tests. Despite, it fits in the range of 5% to 21% of other epidemiological prevalence data (see Table 6). The results demonstrated that the self-reported prevalence of uterine fibroids increased by age, reaching 14.1% in the age group of 40+ years. Findings are consistent with other studies [12,14,15]. Besides, the data showed that some women in the twenties already received a diagnosis of uterine fibroids, though prevalence numbers are rather low (1.8%). The mean age at diagnosis is very similar across all countries and ranges between 33.5 and 36.1 years, which is somehow surprising, considering that the countries differ greatly in getting access to the health care system and in their health care provision.

**Table 6:** Comparison of prevalence data from other studies

First Author	Year	Sample size	Study population	Diagnostic test	Prevalence
Laughlin [10]	2009	n = 4,271	women in the first trimester of pregnancy	transvaginal ultrasonography	10.7% (95%CI: 8.5-13.6%)

Chen [11]	2001	n = 3,174	women aged 17-44 years undergoing tubal sterilization	laparascopy	10.0%*

Borgfeldt [12]	2000	n = 335	asymptomatic women aged 25-40 years	transvaginal ultrasonography	5.4% (95%CI: 3.0-7.8%)

Marino et al. [13]	2004	n = 341	premenopausal women aged 30-60 years	transvaginal ultrasonography	21.4%*

Day Baird [14]	2003	n = 1,364	premenopausal women aged 35-49 years	self-report, medical records, ultrasonography	age 35-39 years: 10-15% in white women; 30-40% in black women*

Downes [31]	2010	n = 4,414	adult female respondends (≥ 18 years)	self-report	from 11.7% (France) to 23.6% (Italy)*

* CI was not indicated

### Risk Factors

Results of this study indicate that age is a risk factor for uterine fibroids as diagnosed women with myomas were significantly older than undiagnosed women. This finding is confirmed by other studies [12,17,32]. In contrast, age at menarche did not appear as a risk factor for women with uterine fibroids, which was found in some other research studies [15,22,33]. Furthermore, after adjusting for age, we could not find any difference in the percentage of women who have given birth between women with and without a diagnosis of uterine fibroids, although parity is supposed to decrease the risk of uterine myomas [15,19,22].

### Symptoms

According to our knowledge, this is the first study that systematically investigated self-reported bleeding and pain symptoms of women with a diagnosis of uterine fibroids. Nevertheless, when interpreting the study results it needs to be considered that diagnosed women within the study population rather reflect those cases who got more clinical attention because of their symptoms, whereas asymptomatic myomas often remain undiagnosed. The results showed that women with a diagnosis of uterine fibroids reported more frequently about heavy and prolonged bleedings compared to women without uterine fibroids. Besides, diagnosed women indicated more often to have unpredictable and irregular bleedings, also described as frequent periods that appear more often than just every 24 days or bleedings between periods. These findings are consistent with previous studies [11,26,34]. While the association between uterine bleeding symptoms and myomas is long known, the pathomechanism is not understood yet. Possible causes are venous ectasia resulting from mechanical compression of veins by myomas, or altered function, expression or storage of vasoactive growth factors produced by myomas [1,35].

The findings in this study suggest that apart from uterine bleeding symptoms, women with uterine fibroids suffer more frequently from multiple gynecological pain symptoms than women without a diagnosis of uterine fibroids. Pain symptoms reported more frequently in women with uterine fibroids were: pressure on the bladder, chronic pelvic pain and pain at different time points during the menstrual cycle. In addition, women with myomas more often suffer from painful sexual intercourse. Even after adjusting for age and co-morbidities women with uterine fibroids reported more often multiple pain symptoms compared to women without a diagnosis. The literature examining the relation of uterine myomas and gynecologic pain symptoms in a population-based study is very limited. An Italian study found that in a non-care-seeking population women with uterine fibroids were more likely to report moderate or severe dyspareunia and moderate or severe non-cyclic pelvic pain than women without uterine fibroids, but not moderate to severe dysmenorrhea [27]. The research studies on pain during sexual intercourse are inconsistent: A study from Ferrero et al. (2006) demonstrated that women with uterine fibroids do not have an increased prevalence or severity of deep dyspareunia, whereas Ertunc et al. (2009) found that a potential impairment, mainly because of pain during sexual intercourse, exists in women with myomas [36,37]. The results of this study suggest that uterine fibroids can cause multiple bleeding and pain symptoms, which may have a negative impact on the sexual life of women, their relationship and family as well as work. Nevertheless further research on the symptoms and their impact on life is needed.

### Management of uterine fibroids

The treatment patterns across the 8 countries vary a lot, due to differences in the health care systems as well as due to cultural differences in patients and physicians treatment preferences. The data showed that the percentage of women who receive medical treatment after diagnosis varies broadly, being highest in Brazil (55.8%; 95%CI: 50.7-60.8%) and lowest in Germany (18.5%; 95%CI: 12.7-25.4%). Oral Contraceptives and Painkillers are across all countries the leading medical treatment option to manage bleeding and pain symptoms, although they do not treat the myomas [29]. Furthermore women in France and the UK commonly reported to use an intrauterine system (Mirena^**®**^) and women in Brazil and France indicated a high usage of progestins for the treatment of uterine fibroids. These hormonal therapies are in general commonly prescribed for gynecological indications in those countries. Except for Brazil, more than half of the women have undergone surgery to treat myomas, considering that the study population might reflect rather symptomatic women with uterine fibroids. Still in most countries, hysterectomy is the leading surgical intervention, except for Italy and France. The results are consistent with the European study from Downes et al. (2010) [31]. As expected Hysterectomy rates are highest in the USA, where uterus myoma is the leading diagnosis for this surgery [8]. The lowest number of hysterectomized women was found in Italy, independently if women have received a diagnosis of uterine fibroids or not.

## Conclusions

The study results are consistent with available data and underline that uterine fibroids are a common concern for women in fertile age, especially in the age group of the over 40s. Uterine fibroids can cause multiple bleeding and pain symptoms which might have a negative impact on women's life, influencing their sexual, social and work life. Despite these consequences uterine fibroid data, especially on epidemiology, symptomatology and their impact on women's health are still limited and further research is required.

## Competing interests

DB, CG, MS, and KG are current employees of Bayer Healthcare Pharmaceuticals. AZ was a former employee of Bayer Healthcare Pharmaceuticals who left the company before the time of research. AZ has acted as paid research consultant for Bayer Healthcare Pharmaceuticals.

## Authors' contributions

AZ was involved in the analysis and interpretation of the data and drafting the manuscript. DB, CG, MS, and KG were involved in designing the study, interpretation of the data and drafting of the manuscript. All authors read and approved the final manuscript.

## Pre-publication history

The pre-publication history for this paper can be accessed here:

http://www.biomedcentral.com/1472-6874/12/6/prepub
